# Brazilian National Palliative Care Policy: reflections based on the 2030 Agenda for Sustainable Development

**DOI:** 10.1590/0034-7167.2024770601

**Published:** 2024-09-23

**Authors:** Ana Cláudia Mesquita Garcia, Geovanna Maria Isidoro

**Affiliations:** I Universidade Federal de Alfenas. Alfenas, Minas Gerais, Brazil

The 2030 Agenda for Sustainable Development, an action plan for people, planet and prosperity, is a global action plan adopted by representatives of all 193 United Nations Member States, meeting at United Nations Headquarters, in New York, from September 25 to 27, 2015. By achieving 17 sustainable objectives, the Agenda aims to promote sustainable development that takes into account not only economic aspects, but also social and environmental ones. Among the Agenda’s objectives, the third indicates the need to ensure a healthy life and promote well-being for all at all ages. Through the goals proposed to achieve this objective, the imperative of seeking significant improvements in healthcare service provision stands out, especially in challenging contexts, such as developing countries. In line with these goals, the need to substantially increase health financing and strengthen the recruitment, development, training and retention of healthcare professionals stands out, with a special focus on regions with limited resources. Effective implementation of this goal is crucial to ensuring that all people, regardless of their location or economic background, have access to adequate healthcare.

In the context proposed by the third goal of the 2030 Agenda for Sustainable Development and its respective goals, an important event that recently occurred in the history of Brazilian public health policies stands out: the approval of the Brazilian National Palliative Care Policy by the Ministry of Health. On 31^st^ 2018, Resolution 41 was published, which provides guidelines for the organization of palliative care (PC), in light of integrated continued care, within the scope of the Brazilian Health System (SUS - *Sistema Único de Saúde*)^(1)^. In December 2023, during a meeting of the Tripartite Intermanagers Commission, the creation of the Brazilian National Palliative Care Policy received approval from the Ministry of Health. Then, on May 22, 2024, Ordinance MO/MoH 3,681 of May 7, 2024, which establishes the Brazilian National Palliative Care Policy - within the scope of the SUS (PNCP-SUS - *Política Nacional de Cuidados Paliativos - no âmbito do SUS*), was published^(2)^. PNCP-SUS reflects the commitment to ensuring a healthy life and promoting well-being for everyone, at all ages, by recognizing the importance of providing dignified and compassionate care to people with serious illnesses that significantly impact quality of life.

According to the World Health Organization (WHO), PC are intended to improve the quality of life of patients and their families in the face of potentially fatal diseases, through the prevention and relief of biopsychosocial and spiritual suffering, through early identification, correct assessment and treatment of pain and other distressing symptoms^(3)^. PC is holistic and active care, aimed at individuals of all ages who are experiencing serious suffering related to their health condition (especially those who are close to the end of life), with the aim of improving quality of life of patients, family and caregivers^(4)^. According to a study carried out on the quality of care provided at the end of life in several countries, Brazil ranked 79^th^ in a sample of 81 countries, which indicates the precariousness of end-of-life care in the country^(5)^. The authors state that financial restrictions, lack of recognition of the importance of this care, lack of national strategies for implementing PC, limited integration of PC into the country’s health system and, mainly, the lack of investment in the area are factors that affect the supply of quality PC^(5)^.

Increasing investment in health is an essential step to ensuring that the population has access to adequate care at all stages of life. PC requires financial resources to guarantee quality assistance, covering trained professionals, medications, equipment and adequate infrastructure. By increasing health financing, developing countries can allocate specific resources to PC programs, ensuring that these services are accessible to all, regardless of their socioeconomic status. Hence, what is already foreseen in Resolution 41 of 2018 stands out, indicating that financing for the organization of PC must be the subject of a tripartite agreement, observing the planning and organization of continued care integrated in the Health Care Network (RAS - *Rede de Atenção à Saúde*)^(1)^.

Furthermore, PC requires a multidisciplinary and multidisciplinary team. The recruitment, development and training of such professionals are vital factors in ensuring that there is a qualified workforce capable enough to deal with the complexities of the PC approach. In this regard, continuous training of healthcare professionals, especially in the neediest areas, is crucial for the adequate provision of PC to the population. In accordance with guidelines for the organization of PC in SUS, it is necessary to promote the institution of PC disciplines and programmatic contents in undergraduate and specialization education for healthcare professionals, offering permanent education in PC for health workers in SUS^(1)^. PNPC-SUS will include actions to encourage education in PC with incentives for the training and continuing education of RAS professionals^(2)^. In addition to access to adequate training, creating attractive working conditions and offering continuous development opportunities can improve the retention of healthcare professionals, ensuring sustainable PC provision.

Investing in PC demonstrates commitment to promoting the well-being and quality of life of the population, aligning with the 2030 Agenda fundamental principles, favoring an environment conducive to the establishment of effective care systems. PNCP-SUS is a concrete step towards the 2030 Agenda goals, as it recognizes the critical importance of humanized care and comprehensive care in challenging health situations.
