# Supplementary material for: Brazilian National Palliative Care Policy: reflections based on the 2030 Agenda for Sustainable Development
Source: Rev Bras Enferm. 2024 Sep 23;77(6):e770601. doi: 10.1590/0034-7167.2024770601 (PMC11419683; doi:10.1590/0034-7167.2024770601)
Supplement: Translation

# Translation: Política Nacional de Cuidados Paliativos: reflexões a partir da Agenda 2030 para o Desenvolvimento Sustentável

**DOI:** 10.1590/0034-7167.2024770601pt

**Authors:** Ana Cláudia Mesquita Garcia, Geovanna Maria Isidoro

**Affiliations:** I Universidade Federal de Alfenas. Alfenas, Minas Gerais, Brasil

A Agenda 2030 para o Desenvolvimento Sustentável, um plano de ação para as pessoas, para o planeta e para a prosperidade, é um plano de ação global adotado por representantes de todos os 193 Estados-Membros das Nações Unidas, reunidos na sede das Nações Unidas, em Nova York, de 25 a 27 de setembro de 2015. Por meio do alcance de 17 objetivos sustentáveis, a Agenda visa promover um desenvolvimento sustentável que leve em consideração não apenas aspectos econômicos, mas também sociais e ambientais. Entre os objetivos da Agenda, o terceiro indica a necessidade de assegurar uma vida saudável e promover o bem-estar para todos em todas as idades. Por meio das metas propostas para o alcance deste objetivo, nota-se a imperatividade da busca por melhorias significativas na prestação de serviços de saúde, especialmente em contextos desafiadores, como nos países em desenvolvimento. De acordo com tais metas, destaca-se a necessidade de aumentar substancialmente o financiamento da saúde e fortalecer o recrutamento, desenvolvimento, formação e retenção de profissionais de saúde, com foco especial em regiões com recursos limitados. A implementação efetiva dessa meta é crucial para garantir que todas as pessoas, independentemente de sua localização ou contexto econômico, tenham acesso a cuidados de saúde adequados.

No contexto proposto pelo terceiro objetivo da Agenda 2030 para o Desenvolvimento Sustentável e suas respectivas metas, destaca-se um importante acontecimento ocorrido recentemente na história das políticas públicas brasileiras de saúde: a aprovação da Política Nacional de Cuidados Paliativos pelo Ministério da Saúde. Em 31 de 2018, foi publicada a Resolução nº 41, que dispõe sobre as diretrizes para a organização dos cuidados paliativos (CP), à luz dos cuidados continuados integrados, no âmbito do Sistema Único de Saúde (SUS)^(1)^. Em dezembro de 2023, durante reunião da Comissão Intergestores Tripartite, a criação da Política Nacional de Cuidados Paliativos recebeu o aval do Ministério da Saúde. Então, em 22 de maio de 2024, foi publicada a Portaria GM/MS nº 3.681, de 7 de maio de 2024, que institui a Política Nacional de Cuidados Paliativos - no âmbito do SUS (PNCP-SUS)^(2)^. A PNCP-SUS reflete o compromisso de assegurar uma vida saudável e promover o bem-estar para todos, em todas as idades, ao reconhecer a importância de proporcionar assistência digna e compassiva às pessoas com doenças graves que impactam significativamente a qualidade de vida.

De acordo com a Organização Mundial da Saúde (OMS), os CP destinam-se a melhorar a qualidade de vida dos pacientes e de seus familiares diante de doenças potencialmente fatais, por meio da prevenção e alívio do sofrimento biopsicossocial e espiritual, através da identificação precoce, correta avaliação e tratamento da dor e de outros sintomas angustiantes^(3)^. Os CP são cuidados holísticos e ativos, destinados aos indivíduos de todas as idades que estão vivenciando sofrimento grave relacionado ao seu quadro de saúde (especialmente os que estão próximos do fim de vida), com o objetivo de melhorar a qualidade de vida dos pacientes, familiares e cuidadores^(4)^. De acordo com estudo realizado sobre a qualidade dos cuidados prestados em fim de vida em diversos países, o Brasil assumiu a 79ª posição do *ranking* em uma amostra de 81 países, o que indica a precariedade dos cuidados de fim de vida no país^(5)^. Os autores afirmam que as restrições financeiras, falta de reconhecimento sobre a importância desses cuidados, falta de estratégias nacionais para efetivação dos CP, integração limitada dos CP no sistema de saúde do país e, principalmente, a falta de investimento na área são fatores que afetam a oferta de CP de qualidade^(5)^.

O aumento do investimento na saúde é um passo essencial para garantir que a população tenha acesso a cuidados adequados em todas as fases da vida. Os CP demandam recursos financeiros para garantir uma assistência de qualidade, abrangendo profissionais treinados, medicamentos, equipamentos e infraestrutura adequada. Ao aumentar o financiamento da saúde, os países em desenvolvimento podem destinar recursos específicos para programas de CP, assegurando que esses serviços estejam acessíveis a todos, independentemente de sua condição socioeconômica. Nesse sentido, destaca-se o já previsto na Resolução nº 41 de 2018, indicando que o financiamento para a organização dos CP deverá ser objeto de pactuação tripartite, observando o planejamento e a organização dos cuidados continuados integrados na Rede de Atenção à Saúde (RAS)^(1)^.

Ainda, os CP exigem uma equipe multiprofissional e multidisciplinar. O recrutamento, o desenvolvimento e a formação de tais profissionais são fatores vitais para garantir que haja uma força de trabalho qualificada e suficientemente capaz para lidar com as complexidades da abordagem dos CP. Nesse sentido, a capacitação contínua dos profissionais de saúde, especialmente nas áreas mais carentes, é crucial para a adequada oferta de CP à população. De acordo com as diretrizes para a organização dos CP no SUS, faz-se necessário fomentar a instituição de disciplinas e conteúdos programáticos de CP no ensino de graduação e especialização dos profissionais de saúde, ofertar educação permanente em CP para os trabalhadores da saúde no SUS^(1)^. A PNPC-SUS contemplará ações de estímulo à educação em CP com incentivo à formação e educação continuada de profissionais da RAS^(2)^. Além do acesso à formação adequada, criar condições de trabalho atrativas e oferecer oportunidades de desenvolvimento contínuo pode melhorar a retenção de profissionais de saúde, assegurando uma prestação sustentável de CP.

Investir em CP demonstra compromisso com a promoção do bem-estar e da qualidade de vida da população, alinhando-se aos princípios fundamentais da Agenda 2030, favorecendo um ambiente propício para o estabelecimento de sistemas de cuidados eficazes. A PNCP-SUS é um passo concreto na direção dos objetivos da Agenda 2030, visto que reconhece a importância crítica do cuidado humanizado e da assistência integral em situações de saúde desafiadoras.

# Translation: Política Nacional de Cuidados Paliativos: reflexiones a partir de la Agenda 2030 para el Desarrollo Sostenible

**DOI:** 10.1590/0034-7167.2024770601es

**Authors:** Ana Cláudia Mesquita Garcia, Geovanna Maria Isidoro

**Affiliations:** I Universidade Federal de Alfenas. Alfenas, Minas Gerais, Brasil

La Agenda 2030 para el Desarrollo Sostenible, un plan de acción para las personas, el planeta y la prosperidad, es un plan de acción global adoptado por representantes de los 193 Estados Miembros de las Naciones Unidas, reunidos en la Sede de las Naciones Unidas, en Nueva York, del 25 al 27 de septiembre de 2015. Al alcanzar 17 objetivos sostenibles, la Agenda pretende promover un desarrollo sostenible que tenga en cuenta no sólo los aspectos económicos, sino también los sociales y ambientales. Entre los objetivos de la Agenda, el tercero señala la necesidad de garantizar una vida sana y promover el bienestar para todos en todas las edades. A través de las metas propuestas para alcanzar este objetivo, se advierte el imperativo de buscar mejoras significativas en la prestación de servicios de salud, especialmente en contextos desafiantes, como el de los países en desarrollo. En línea con estos objetivos, destaca la necesidad de incrementar sustancialmente el financiamiento de la salud y fortalecer el reclutamiento, desarrollo, capacitación y retención de profesionales de la salud, con especial enfoque en las regiones con recursos limitados. La implementación efectiva de este objetivo es crucial para garantizar que todas las personas, independientemente de su ubicación o situación económica, tengan acceso a una atención sanitaria adecuada.

En el contexto propuesto por el tercer objetivo de la Agenda 2030 para el Desarrollo Sostenible y sus respectivas metas, se destaca un hecho importante ocurrido recientemente en la historia de las políticas públicas de salud brasileñas: la aprobación de la Política Nacional de Cuidados Paliativos por parte del Ministerio de Salud. El 31 de 2018, se publicó la Resolución nº 41, que establece directrices para la organización de los cuidados paliativos (CP), en el marco de la atención continuada integrada, en el ámbito del Sistema Único de Salud (SUS - *Sistema Único de Saúde*)^(1)^. En diciembre de 2023, durante una reunión de la Comisión Tripartita Intergestores, la creación de la Política Nacional de Cuidados Paliativos recibió el visto bueno del Ministerio de Salud. Luego, el 22 de mayo de 2024, fue publicada la Ordenanza GM/MS nº 3.681, de 7 de mayo de 2024, que establece la Política Nacional de Cuidados Paliativos - en el ámbito del SUS (PNCP-SUS - *Política Nacional de Cuidados Paliativos - no âmbito do SUS*)^(2)^. El PNCP-SUS refleja el compromiso de garantizar una vida saludable y promover el bienestar para todos, en todas las edades, al reconocer la importancia de brindar atención digna y compasiva a las personas con enfermedades graves que impactan significativamente la calidad de vida.

Según la Organización Mundial de la Salud (OMS), los CP tienen como objetivo mejorar la calidad de vida de los pacientes y sus familias ante enfermedades potencialmente mortales, mediante la prevención y el alivio del sufrimiento biopsicosocial y espiritual, mediante la identificación temprana, la evaluación y el tratamiento correctos del dolor y otros síntomas angustiantes^(3)^. El CP es una atención holística y activa, dirigida a personas de todas las edades que atraviesan un sufrimiento grave relacionado con su estado de salud (especialmente aquellos que se encuentran cerca del final de la vida), con el objetivo de mejorar la calidad de vida de los pacientes, sus familiares y sus cuidadores^(4)^. Según un estudio realizado sobre la calidad de los cuidados prestados al final de la vida en varios países, Brasil ocupó el puesto 79 en una muestra de 81 países, lo que indica la precariedad de los cuidados al final de la vida en el país^(5)^. Los autores afirman que las restricciones financieras, la falta de reconocimiento de la importancia de este cuidado, la falta de estrategias nacionales para implementar el CP, la limitada integración del CP al sistema de salud del país y, principalmente, la falta de inversión en el área son factores que inciden en la oferta de CP de calidad^(5)^.

Incrementar la inversión en salud es un paso esencial para garantizar que la población tenga acceso a una atención adecuada en todas las etapas de la vida. El CP requiere recursos financieros para garantizar una asistencia de calidad, que abarque profesionales capacitados, medicamentos, equipos e infraestructura adecuada. Al aumentar el financiamiento de la salud, los países en desarrollo pueden asignar recursos específicos a programas de CP, asegurando que estos servicios sean accesibles para todos, independientemente de su estatus socioeconómico. En este sentido, destaca lo ya previsto en la Resolución n° 41 de 2018, indicando que el financiamiento para la organización del CP debe ser objeto de un acuerdo tripartito, observar la planificación y organización de la atención continuada integrada en la Red de Atención a la Salud (RAS - *Rede de Atenção à Saúde)* ^(1)^.

Además, el CP requiere de un equipo multidisciplinar y multidisciplinar. La contratación, el desarrollo y la formación de dichos profesionales son factores vitales para garantizar que exista una fuerza laboral cualificada y capaz de afrontar las complejidades del enfoque de CP. En este sentido, la formación continua de los profesionales sanitarios, especialmente en las zonas más necesitadas, es crucial para la adecuada prestación de CP a la población. De acuerdo con las directrices para la organización del CP en el SUS, es necesario promover la institución de disciplinas y contenidos programáticos en CP en la educación de pregrado y especialización de los profesionales de la salud, ofreciendo educación permanente en CP para los trabajadores de la salud del SUS^(1)^. El PNPC-SUS incluirá acciones para incentivar la educación en CP con incentivos para la formación y educación continua de los profesionales de la RAS^(2)^. Además del acceso a una formación adecuada, la creación de condiciones laborales atractivas y la oferta de oportunidades de desarrollo continuo pueden mejorar la retención de los profesionales sanitarios, garantizando un suministro sostenible de CP.

Invertir en CP demuestra el compromiso de promover el bienestar y la calidad de vida de la población, alineándose con los principios fundamentales de la Agenda 2030, favoreciendo un entorno propicio para el establecimiento de sistemas de cuidados eficaces. El PNCP-SUS es un paso concreto hacia los objetivos de la Agenda 2030, ya que reconoce la importancia crítica de la atención humanizada y la asistencia integral en situaciones de salud desafiantes.
